# Spatio-Frequency Decoupled Weak-Supervision for Face Reconstruction

**DOI:** 10.1155/2022/5903514

**Published:** 2022-09-22

**Authors:** Yanyan Li, Weilong Peng, Keke Tang, Meie Fang

**Affiliations:** Guangzhou University, Guangzhou, China

## Abstract

3D face reconstruction has witnessed considerable progress in recovering 3D face shapes and textures from in-the-wild images. However, due to a lack of texture detail information, the reconstructed shape and texture based on deep learning could not be used to re-render a photorealistic facial image since it does not work in harmony with weak supervision only from the spatial domain. In the paper, we propose a method of spatio-frequency decoupled weak-supervision for face reconstruction, which applies the losses from not only the spatial domain but also the frequency domain to learn the reconstruction process that approaches photorealistic effect based on the output shape and texture. In detail, the spatial domain losses cover image-level and perceptual-level supervision. Moreover, the frequency domain information is separated from the input and rendered images, respectively, and is then used to build the frequency-based loss. In particular, we devise a spectrum-wise weighted Wing loss to implement balanced attention on different spectrums. Through the spatio-frequency decoupled weak-supervision, the reconstruction process can be learned in harmony and generate detailed texture and high-quality shape only with labels of landmarks. The experiments on several benchmarks show that our method can generate high-quality results and outperform state-of-the-art methods in qualitative and quantitative comparisons.

## 1. Introduction

3D face reconstruction, which aims to recover 3D face shapes from a single image or multiple-view images, has been widely applied to face recognition [1], face animation [2], and artistic editing [3]. Traditional methods involve complex and costly optimization for accurate reconstruction [4–7]. Since deep learning has significant advantages of nonlinear fitting ability on complex tasks [8–12], there is an increasing interest in reconstructing 3D faces from a single image using deep convolutional neural networks [13–17]. However, the reconstruction accuracy is seriously affected by the challenging cases, e.g., various illumination poses, occlusions, etc.

Generally, deep learning-based methods could be roughly divided into families of supervised learning [16, 18, 19], unsupervised learning [20–22], and weakly supervised learning [13, 23]. For supervised learning, 3D ground-truth face data are needed as supervision information, but a large amount of label data are not easily accessible. For compromise, existing methods usually use 3DMM parameters [18] or traditional methods [19, 24] to synthesize 3D shapes as ground-truth face data, which limits the precision of reconstruction. Unsupervised and weakly-supervised learning overcome the weakness of relying on 3D ground-truth data and learning the reconstruction process based on image data with only labeled landmarks if necessary. Classically, based on the 3DMM model prior, Deng et al. [13] devised a robust loss function combining image-level and perception-level information as weakly supervised information to improve 3D face reconstruction. However, it could not handle the wrong texture when the face is occluded. Feng et al. [18] abandoned the 3DMM model and regressed the 3D shape from the network straightly, but their supervision data are still based on the 3DMM fitting, which has limitations.

In our opinion, a key reason for the lack of high reconstruction accuracy is that the commonly used CNNs approach only considers spatial loss [13, 25, 26], e.g., landmark loss and pixel loss in the spatial domain, while ignoring the impact of frequency. Some studies have shown that DNNs tend to synthesize frequencies in order from low to high [27–29]. So it is hard to urge neural networks to learn the inconspicuous frequency of images and recover them with merely spatial loss [16] since spatial loss focuses on point-wise value and spatial associations but does not pay enough attention to harmony in the frequency domain [30].

Based on the abovementioned points, we proposed a spatio-frequency decoupled weak-supervision approach for 3D face reconstruction to address the unreality issue. We first use a convolutional neural network (ResNet-50) to regress 3DMM coefficients and render parameters. And then, we build the weakly supervision between the input and the re-rendered face image. Not limited to spatial domain loss covering image-level and perceptual-level loss, frequency spectrums are also separated from image pairs to measure the gap in the frequency domain based on differentiable discrete Fourier transformation. We devise the patch-level frequency loss based on spectrum-wise weighted Wing loss to capture further inconspicuous frequency affecting reality. In particular, the loss motivates the network to learn detailed textures and avoids the adverse effects of occlusion. Experiments show that our method can generate high-quality results and outperform several state-of-the-art methods in qualitative and quantitative comparisons on several benchmarks. To summarize, this paper makes the following contributions:

- We propose a spatio-frequency decoupled weak supervision method for 3D reconstruction with high-fidelity textures from a single in-the-wild image.
- We propose a patch-based spectrum-wise weighted Wing loss in the frequency domain to improve the robustness of texture reconstruction to occlusion and the reality of the re-rendered image.

## 2. Related Work

### 2.1. 3D Face Detail Reconstruction

***Geometry Reconstruction***. The 3DMM [31] makes it possible to recover the facial shape from a single image by regressing 3DMM face shape parameters. Some studies [14, 32] reconstructed a rough shape using the 3DMM in the first stage and then refined the shape by imposing some spatial domain constraints, e.g., asymmetric Euclidean loss [32] and identity consistency loss [14]. The other methods [26, 33] used a collaborative approach by employing a synergy process between 3DMM coefficients and 3D face landmarks [33] or an occlusion segmentation network [26]. These approaches narrow the error in the spatial domain to synthesize more realistic facial geometry with 3DMM. But 3DMM works well in the low-frequency domain, neglecting the critical frequency information that determines the realism. In contrast, we aim to capture the key frequency in the frequency domain.

***3D Re-Renderable Modeling***. 3D Re-renderable modeling makes the process of mapping a 3D face model to a 2D portrait image [21, 34–37]. These methods decompose a single face image into reflectance, geometry, and lighting and then render the face image by changing the lighting and fixing the geometry and reflectance [38]. Yamaguchi et al. [36] developed a deep learning method to estimate high-resolution facial reflectance and normal. However, they could not re-render the whole face image while leaving out the eye, teeth, and hair regions. Dib et al. [34] introduced ray tracing for face reconstruction within an optimization-based framework to make the re-rendered faces robust to lighting conditions. But the quality of their reconstruction is still influenced by the initialization landmarks. Yang et al. [37] proposed a novel, detailed illumination representation to disentangle facial texture and lighting, resulting in high-fidelity textures even with in-the-wild images. Their results are good but also decoupled in the spatial domain. Different from them, our method decouples illumination and albedo in the frequency domain to obtain an anti-occlusion, anti-illumination, and re-renderable face image.

### 2.2. Frequency Domain Studies of Neural Networks

Several studies [27, 28] have begun to use Fourier analysis to explore the neural network training process and found a learning bias of neural networks towards low-frequency components. Moreover, F-Principle [29] showed that the frequency fitting priority is different throughout the training process, usually from low to high. Therefore, when using CNN for reconstructing a 3D face shape, the network always avoids high-frequency components, which will cause the reconstructed 3D face to be too smooth, and some details cannot be reconstructed.

Recently, Jiang et al. [30] introduced frequency domain information into image synthesis to improve the effect of image synthesis by guiding the network to synthesize hard frequencies that are difficult to synthesize. Although the paper demonstrated the influence of frequency domain information on image synthesis, few studies have explored the effect of frequency in 3D face reconstruction. Wang et al. [39] are the first to introduce the concept of frequency domain into 3D face reconstruction. It enhances self-supervised learning by adding low-frequency albedo information to guide the network for generating intact albedos. However, the albedo model is still a linear subspace model that concentrates on low-frequency, failing to synthesize high-frequency information during training and address the frequency bias problem of DNN training. Our method aims to narrow the frequency gap during the training, i.e., by transforming the image from the spatial domain to the frequency domain based on a differentiable 2D Fourier transform and then reconstructing more detailed 3D faces and albedo.

### 2.3. Wing Loss

Wing loss is a supervised function for face landmark alignment proposed by Feng et al. [40]. After analyzing L1 loss, L2 loss, and smooth L1 loss function empirically and theoretically, they found that large errors easily dominate the step size of these loss functions so that some outliers may mislead the network during training. So they proposed Wing loss to improve the resistance to large errors and the ability to amplify small and medium-scale errors during neural network training.

In 3D face reconstruction work, the importance of high-frequency and low-frequency components are different in an image, and then it is also different in the difficulty of fitting them via neural networks. In the early stage of training, the frequency gap between the input and the re-rendered image is large and becomes small in the middle and later stages of training. However, the error in pixel level may be large when occlusion occurs in the image, even though the frequency error can be very small. To narrow the gap further and improve the reconstructed face's accuracy, we use Wing loss to solve the problem. Inspired by the Wing loss's variant [41], we adjust the spectrum-wise weighting experimentally so that the differences could be suppressed even at the tiny frequency error by amplifying the spectrum-wise error. In this way, the effect brought by occlusion frequencies can be significantly alleviated.

## 3. Method

### 3.1. Preliminaries

Our approach regresses the shape and texture coefficients of the 3DMM model to reconstruct the 3D face shape, which is then rendered onto a 2D plane, using spatial and frequency domain information as weak supervision signals to assist the network training. We will introduce several foundation works involved in the procedure, including the 3DMM, illumination, and camera models.

***Face prior model***. 3DMM [31] is our face prior model for reconstructing face shape and texture based on principal component analysis (PCA). As the original 3DMM could not express facial expressions, we improved the 3DMM model by fusing the expression bases **A**_exp_ built from Face-Warehouse [42]. At last, the model is defined as:

(1) $$\begin{array}{c} S\left(\alpha ,\beta \right)=\bar{S}+A_{id}\alpha +A_{\exp}\beta ,\\ T\left(\delta \right)=\bar{T}+A_{t}\delta , \end{array}$$

where $\bar{S}$ and $\bar{T}$ represent the mean shape and texture, **A**_*id*_ and **A**_**t**_ are the PCA bases with a neutral expression. **α** ∈ ℝ^80^, **β** ∈ ℝ^64^ and **δ** ∈ ℝ^80^ are the shape, expression, and texture parameters to be regressed in our model.

***Camera model.*** We use a perspective model as the camera model. It first converts any vertex **v** on **S** to a new position $\hat{v}$ under the camera coordinate system with orthogonal rotation **R** ∈ *SO*(3) and translation **t** ∈ ℝ^3^. And then ${\hat{v}}_{i}$ is projected to point **u** in an image plane. In particular, we set an empirical focal in the camera to display the 2D face. On the whole, there are six parameters in the perspective model.

***Illumination model***. Assuming the human face is a Lambert surface, we use the spherical harmonic (SH) function to represent scene illumination and then compute the radiosity of the vertex [43]. With the surface normal **n**_*i*_ at ${\hat{v}}_{i}$, the radiosity *I*_*i*_ related to the pixel can be represented by the SH illumination model with three bands:

(2) $$\begin{array}{c} I_{i}\left(l\right)=t_{i}\sum_{j=1}^{9}l_{j}H_{j}\left(n_{i}\right), \end{array}$$

where *t*_*i*_ is one channel of texture at **v**_*i*_ on **T**, and **l** is channel-wise control coefficients, {*H*_*j*_} is orthogonal bases in spherical harmonic function. Generally, the SH model can accurately estimate the illumination information in different environments without estimating the direction of the light source, which greatly simplifies the illumination estimation.

***Unsupervised learning reconstruction***. Under an unsupervised schema, all the unknown parameters are predicted as Θ ∈ ℝ^257^ that consists **α**, **β**, **δ**, **R**, **t** and {**l**_*i*_}_*i*∈{*r*, *g*, *b*}_ by a neural network for a given face image *I*, firstly. And then Θ is applied to a differentiable image formation layer to generate a new rendered image *I*′. The shape and texture could be learned by supervising *I*′ with input *I*:

(3) $$\begin{array}{c} \min\text{Dist}\left(I,I^{\prime }\left(\Theta \right)\right),\text{with}\Theta =\text{NeurlNet}\left(I\right). \end{array}$$

Under the formulation, skin masks [44, 45], and weak supervision with landmarks [46, 47] could be introduced to learn high-quality face.

### 3.2. Framework of Spatio-Frequency Decoupled Weak-Supervision

**Figure 1:**
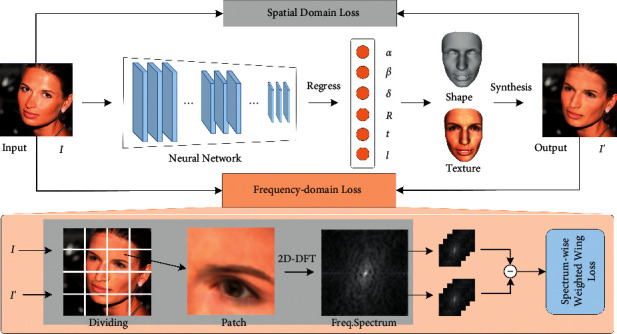
Overview of our approach. Our network is a weak-supervision network that considers both spatial and frequency-domain loss. The entire architecture feeds a single 2D image into the convolutional neural network (ResNet-50) to regress the 3DMM coefficients **α**, **β**, **δ** and rendering parameters *I*, *p*. With the parameters, we can reconstruct the 3D shape and texture, and synthesize the re-rendered image. A spectrum-wise weighted Wing loss is devised for fine fitting in the frequency domain.

We will introduce spatio-frequency decoupled weak-supervision into equation (3). In our framework, the learning process is applied with supervision in both spatial domain and frequency domain, seeing in Figure 1. Firstly, a convolutional neural network (ResNet-50) is used to regress the parameters of shape, texture, pose, and illumination from face image *I*. Then it outputs rendered image *I*′ according to differentiable analytic synthesis. The spatial and frequency-domain losses are applied during the training stage to learn high-quality shapes and textures.

#### 3.2.1. Spatial Domain Loss

***Landmark-level***. The alignment of facial landmarks is the alignment of high-level semantics between pixels of face images. To supervise the network, we usually project the shape we get abovementioned into the 2D image and minimize the difference between its 68 landmarks *K*_*i*_^*p*^ and the ground-truth 68 landmarks *K*_*i*_^*g*^. Moreover, we assign different weights *w*_*i*_ to different face parts. The landmark loss is defined as:

(4) $$\begin{array}{c} L_{lmk}=\frac{1}{N}\sum_{i=1}^{N}w_{i}{\left\|K_{i}^{g}-K_{i}^{p}\right\|}^{2}. \end{array}$$

***Image-level***. Based on equation (3), we build the image-level loss according to the photometric discrepancy between the original image *I* and the reconstructed image *I*′. To weaken the harmful effect brought by hair and face decoration, a skin mask is introduced to guide the loss as follows:

(5) $$\begin{array}{c} L_{\text{img}}=\frac{\left\|M⊙I-M⊙I^{\prime }\right\|}{\left\|M\right\|}. \end{array}$$

***Perceptual-level***. Some traditional methods use low-level information as the supervision information of the network, which results in smooth output images, so the appropriate selection of a layer of output features input perceptual loss function can enhance the details. Influenced by recent work [13], we also use a pretrained face recognition network to fit this deep level of information during training. Perceptual loss is defined as:

(6) $$\begin{array}{c} L_{\text{per}}=1-\frac{f\left(I\right)f\left(I^{\prime }\right)}{\left\|f\left(I\right)\right\|\left\|f\left(I^{\prime }\right)\right\|}, \end{array}$$

where *f*(·) denotes deep feature encoding.

**Figure 2:**
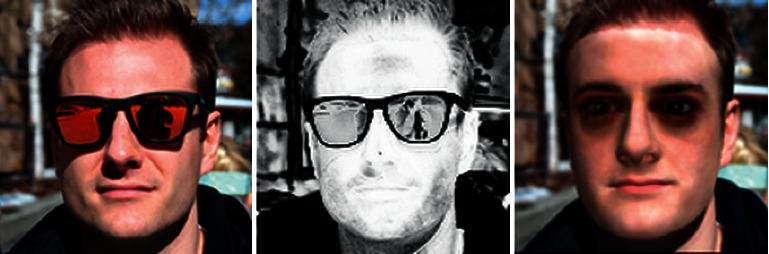
The shadow problem brought by using only spatial domain loss: in the mask map (middle), we found that when the occlusion color is complex, the mask is correspondingly not good, so it will lead to the phenomenon of “under-eye dark circle” (right).

***The problem brought by spatial loss***. Image-level loss learns uncertain texture when severe occlusions exist on the face.  Figure 2 shows the output texture has black eyes when wearing glasses on the face. The reason is that DNN learns weights from high frequency to low frequency during the process of fitting images, but it is challenging to work in harmony without explicit guidance on the frequency domain [27].

#### 3.2.2. Frequency-Domain Loss

Since the spatial domain loss could not handle the issue of facial occlusion well, we propose to use the frequency domain loss to alleviate it. Inspired by [30], we convert the input image and the rendered output image into their frequency representations and model the supervision between them.

***Frequency representation***. The representation in the frequency domain can be implemented by differentiable discrete Fourier transformation (DFT) [30], being formulated by:

(7) $$\begin{array}{c} F\left(u,v\right)=\sum_{x=0}^{M-1}\sum_{y=0}^{N-1}f\left(x,y\right)\cdot e^{-i2\pi \left(ux/M+vy/N\right)}. \end{array}$$

**Figure 3:**
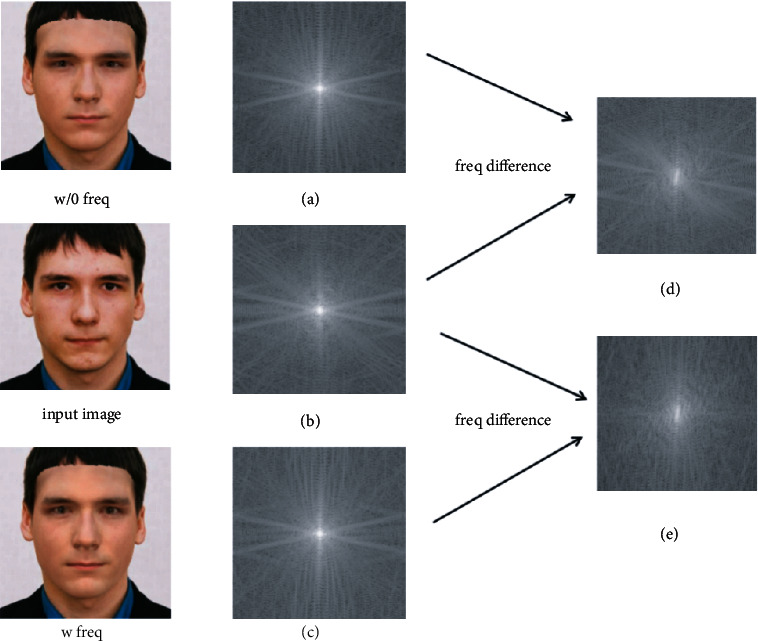
The difference in frequency spectrum with and without frequency supervision: (b) is the input image's spectrum. (a) and (c) are the frequency spectrums of the re-rendered images without and with frequency supervision; (d) and (e) are the difference between the two re-rendered spectrums, respectively.

Figure 3 shows that there is a certain gap between the frequency spectrums with and without frequency supervision. The frequency difference between Figures 3(a) and 3(b) and the frequency difference between Figures 3(c) and 3(b) are reflected in Figures 3(d) and 3(e), respectively. It is not difficult to find that after performing the differential calculation in the frequency domain, the generated frequency spectrum by our frequency domain supervision is closer to the original input image. Therefore, using the frequency domain loss, a supervision signal to assist the reconstruction of 3D faces, the network can synthesize frequencies that are not easy to synthesize effectively.

***Frequency-based wing loss***. We devise a loss function based on frequency representation for retrieving the missing frequencies in the re-rendered image.

Moreover, to learn more subtle changes in the frequency domain, the Wing loss [40] is adopted to design the frequency loss based on local patches divided from images:

(8) $$\begin{array}{c} L_{freq}=\frac{\sum_{u=0}^{M-1}\sum_{v=0}^{N-1}\sum_{p=0}^{P-1}Wing\left(F^{\left(p\right)}\left(u,v\right)-F^{\prime \left(p\right)}\left(u,v\right)\right)}{MNP}, \end{array}$$

where *M* and *N* are height and width of image, and *P* is the number of patches. *F*(*u*, *v*) is the spatial frequency value at the spectrum coordinate (*u*, *v*) of the input image *I*, and *F*′(*u*, *v*) is that of re-rendered image *I*′. The advantage of Wing loss is that the gradient keeps high even at a minimal error. Thus, the low frequency that determines the realism of rendering could be enlarged to improve the reconstruction quality.

**Figure 4:**
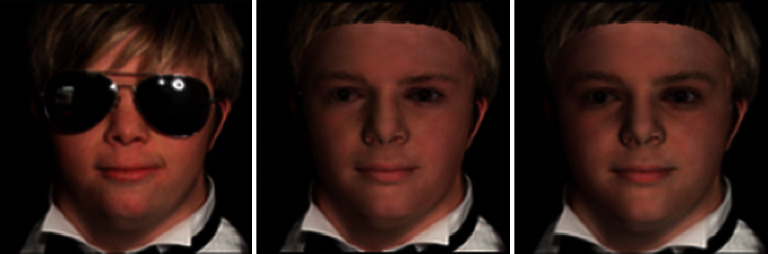
From left to right: the input image, the image rendered using the constant weighting, and the image rendered using the spectrum-wise weight. The right image is much more colorful than the middle one.

***Spectrum-wise weighting***. Under original Wing loss, the weights for different frequencies are equal and constant. In our design, we hope to pay more attention to the high-frequency part and less to the low-frequency. Therefore, we propose spectrum-wise weights for the frequency-based Wing loss, defined as:

(9) $$\begin{array}{c} \text{Wing}\left(y,u,v\right)=\left\{\begin{array}{cc} w\left(u,v\right)\text{ln}\left(\frac{1+\left|y\right|}{\epsilon }\right), & \text{if}\left|y\right|<w\left(u,v\right),\\ \left|y\right|-C, & \text{otherwise,} \end{array}\right. \end{array}$$

where *y* = Δ*a* + Δ*b* · *i*, and *C* = *w*(*u*, *v*) − *w*(*u*, *v*)ln(1 + *w*(*u*, *v*)/*ϵ*). Δ*a* and Δ*b* are the difference of real and imaginary parts, respectively, between *F*(*u*, *v*) and *F*′(*u*, *v*). And *w*(*u*, *v*) is also the spectrum-wise threshold between a linear and nonlinear part of the wing curve, which is learned adaptively during training. As Figure 4 shows, spectral weighted Wing loss gets more saturated and closer to the actual face texture. What's more, different from Figure 2, the neural network no longer only uses simple pixel-level supervision information but also the supervision in the frequency domain. It has a specific resistance to the phenomenon of dark circles under the occlusion of sunglasses.

## 4. Experimental Results

***Training data pipeline***. In terms of training set, we get∼320 k face images from CelebA [48], FFHQ [49] and Multi-PIE [50]. Then we use the method of [51] to align and crop facial images for model input.

***Detailed setting***. We follow the method of [13] which trained a naïve Bayes classifier with Gaussian mixture model on a skin image dataset from [52] to generate the mask used in image-level loss, and then preprocess the training set. We use the Adam optimizer for ResNet-50 [53] that predicts Θ and its initial learning rate is set to 1e − 4. The total loss converges after about 200 K iterations.

### 4.1. Qualitative Evaluation

**Figure 5:**
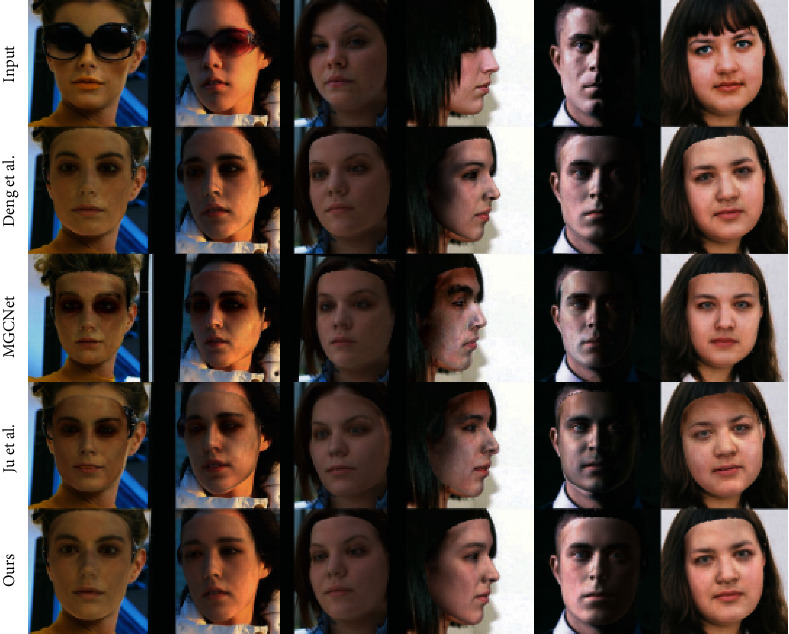
Comparison with Deng et al. [13], MGCNet [54]. and Ju et al. [55] Our re-rendered images are better in the details and are more consistent with the input image. The images are from AFLW2000 [17].

**Figure 6:**
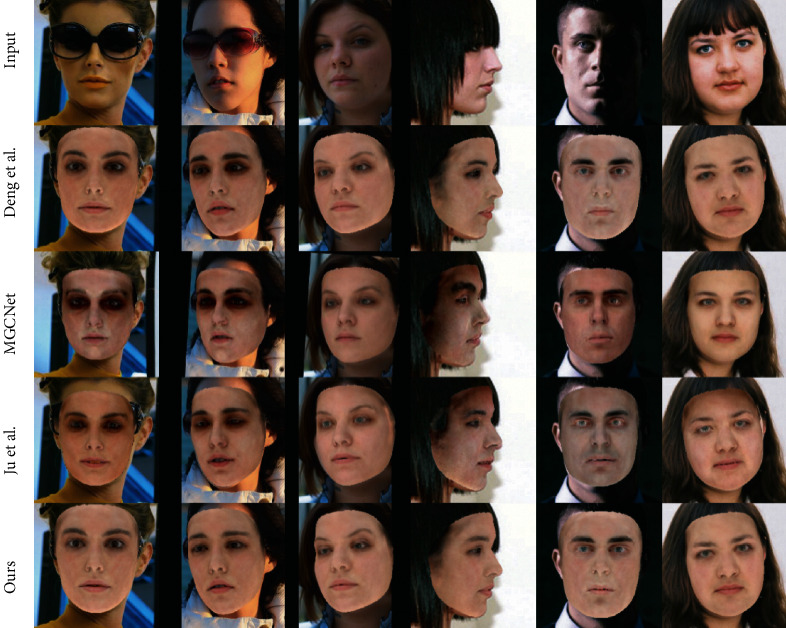
Comparison with Deng et al. [13], MGCNet [54] and Ju et al. [55]. Without illumination, the textures synthesized by our method more closely match the original images and are resistant to occlusion colors. The images are from AFLW2000 [17].

Figures 5 and 6 shows the re-rendered images and textures overlayed on original images, respectively, by comparing the methods [13, 54, 55] on the AFLW2000 dataset [17]. Ju et al. [55] used GAN to repair the occlusion images after obtaining the textures from 3DMM model, which did not use the image-level loss but the adversarial loss. Deng et al. [13] used a robust loss including pixel loss, for 3D face reconstruction. MGCNet [54] is a multi-view-based 3D face reconstruction method. It shows that our texture does not have black shadows in the presence of occlusions like hair, glasses, and poor lighting. Moreover, our method can also help the network reconstruct more detailed faces, such as the reconstruction of the eyes in the third column of Figure 5.

**Figure 7:**
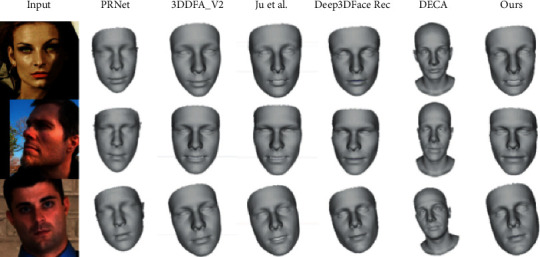
Comparison to other recent reconstruction methods, from left to right: PRNet [18], 3DDFA_V2 [56], Ju et al. [55], Deep3DFaceRec [13], DECA [25] and Our method. The images are from AFLW2000 [17].

Figure 7 shows our results in shape compared to recent methods [13, 18, 25, 55, 56]. The 3D face shape reconstructed by our method is very close to the input image in the case of poor illumination and large occlusions. And we could find that our results are more finely synthesized on the eyes and mouth relative to Deep3DFaceRec [13], with a slight advantage.

### 4.2. Quantitative Comparison

#### 4.2.1. FaceScape Benchmark

FaceScape benchmark [16] is an all-sided evaluation method that considers various poses, expressions, environments, and focal lengths to evaluate the accuracy of single-view face 3D reconstruction. It includes two parts of data: FS-Wild data and FS-Lab data. The FS-Wild data consists of 400 face images of 400 synthesized subjects, each with a reference 3D face model, and is divided into four groups according to the camera orientation and the face orientation (0°–5°, 5°–30°, 30°–60°, and 60°–90°). The FS-Lab renders 330 images using the 20 detailed 3D models with three different focal lengths: 1200 (long focal), 600 (middle focal), 300 (short focal), and eleven different camera locations, which one camera at exact front 0°, eight cameras deflecting 30° and two cameras deflecting 60°.

We compared our methods with publicly available methods, i.e., Deep3DFaceRec [13], MGCNet [54], DECA [25], 3DDFA_V2 [56], PRNet [18], FaceScape_deep [16], and UDL [20]. Since the FaceScape benchmark has 3D ground-truth data, Chamfer Distance (CD) measures the error between the predicted and ground-truth mesh. Mean normal error (MNE) measures the intersection of the valid region distance between the predicted normal map and ground-truth normal map, which are obtained from the corresponding mesh rendered in the cylindrical coordinate. The complete rate (CR) measures the completeness of the reconstruction results.

#### 4.2.2. Comparison on FS-Wild Datasets

**Figure 8:**
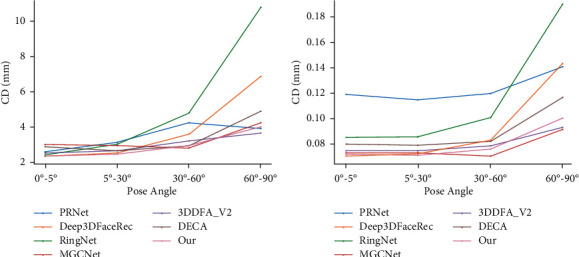
Charts to visualize the quantitative evaluation on FS-wild dataset: (a) Chamfer distance and (b) Mean normal error.

Figure 8 shows the values of CD and MNE under different pose angles in the FS-Wild datasets. The Chamfer distance measured shows the overall error distance in Figure 8(a). Our method performs well in frontal and side views, especially for the frontal and small pose angle views. The results of MNE are shown in Figure 8(b), although, we are not as good as Deep3DFaceRecon [13] at a small pose angle, our effect is much better than as the face angle increases. Furthermore, in the case of large pose angle, our performance is third only to MGCNet [54] which used 3D-ground truth supervision, and 3DDFA_V2 [56] which, we exceed its performance on small pose angle.

#### 4.2.3. Comparison on FS-Lab Datasets

This section reports the values of CD, MNE, and CR of several methods at different pose angles on FS-Lab datasets.

In Table 1, We can see that most methods perform well in the frontal view but severely degrade in the side view. And our method is not only relatively stable for side view but also has the best performance results.

In addition, it is worth noting that CR measures the completeness of the reconstruction results, which is defined as: *η*=*S*(*P*_*p*_∩*P*_*g*_)/*S*(*P*_*g*_). The position map *P*_*p*_ and *P*_*g*_ are the predicted mesh and the ground-truth mesh rendering in the cylindrical coordinate. *S*(*P*) is the function that returns the area of the position map *P*. Limited by the 3DMM model, our method uses the BFM model, excluding the ear and neck region, to reconstruct the actual face area as much as possible. However, 3DDFA_V2 [56] used the MFF [57] algorithm to fit 3DMM parameters and further completed the model to a complete head model with ears and neck. And the DECA [25] reconstructed the entire head with the FLAME model. Obviously, our 3D reconstruction is comparable with other methods.

### 4.3. Ablation Study

To verify the effectiveness of our frequency-domain loss, we perform ablation experiments on Now datasets [58] and AFLW2000-3D [17] datasets.

**Figure 9:**
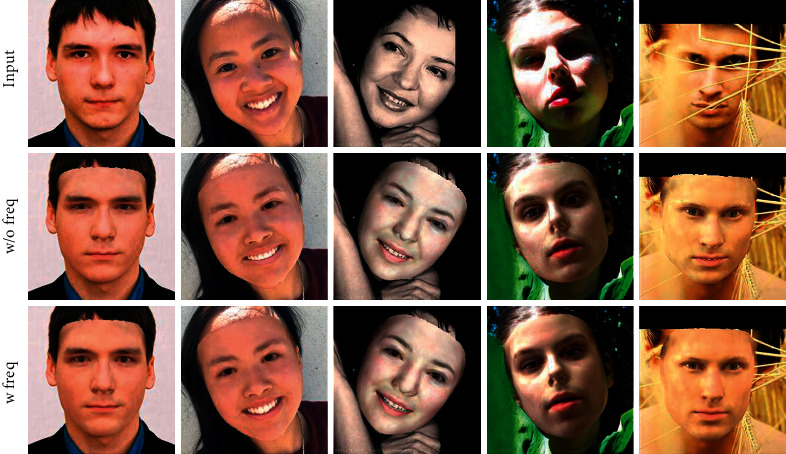
Ablation study on the proposed frequency-domain loss. The frequency-domain method has a better synthesis effect on the eyes, lips, etc. From up to down: the input, the result without frequency loss, and that with frequency loss.

***Frequency domain loss***. To show the importance of our frequency-domain loss, we train our model with and without frequency-domain loss and compare the results.  Figure 9 shows that the frequency loss can assist the convolutional neural network in synthesizing some details that are not easy to synthesize. More detailed face features can be captured in the areas of the eyes, mouth, etc. Moreover, the reconstruction is also very accurate when the face is occluded.

**Figure 10:**
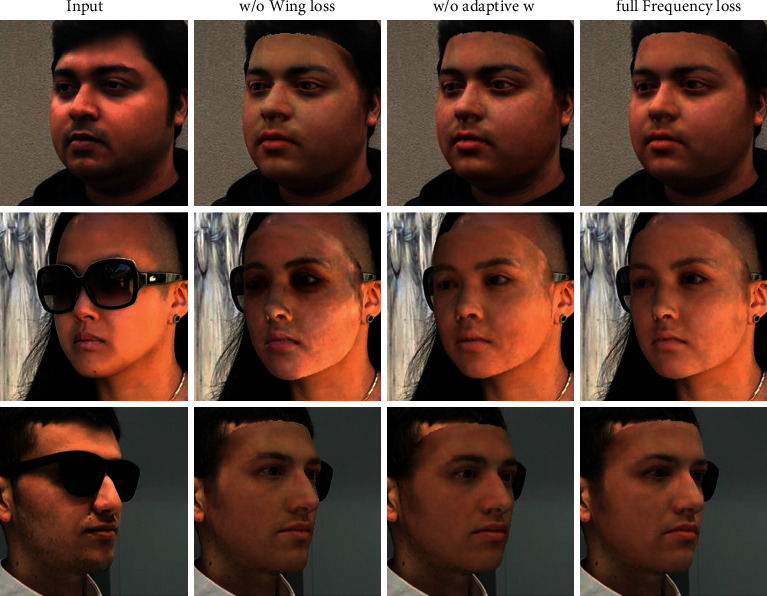
Ablation study of each key component for the frequency loss, i.e., Wing loss and spectrum-wise weight. From left to right: the input, with *l*_2_ loss (w/o Wing loss), the re-rendered results with constant weighting (w/o adaptive w), and with full loss. The input samples are from [58].

***Wing loss and spectrum-wise weighting***.  Figure 10 shows that the full patch-based spectrum-wise weighted Wing loss achieves the best performance. If we use *l*_2_ loss instead of Wing loss, it could not amplify some smaller frequencies error, resulting in underfitting the reconstructed frequency for face synthesizing. Thus, the facial texture is not uniform enough on the whole face.

Moreover, it is noteworthy that the occlusion part will be overfitted when the face is occluded. Wing loss can remove shadows caused by occlusion for two reasons. On the one hand, we use the generated mask to make the network pay little attention to the occlusion part. On the other hand, we use spectrum-wise weighted Wing loss to amplify the error of the high-frequency part and suppress the large frequency difference. Generally, the mask could not perfectly cover some complex, occluded parts of the face. If we only used the pixel-level loss, the color of the occluder would still be fitted. Actually, in the later stage of network training, the frequency gap of occluded parts between the input and reconstructed image will be much larger than that of the unoccluded parts. Spectrum-wise weighted Wing loss guides the network to synthesize frequencies that are not easy to synthesize rather than the shadow parts. Thereby, the reconstruction can be learned in harmony, and the shadow effect caused by occlusion is alleviated to a certain extent.

On the contrary, if we use Wing loss with fixed weighting, it ignores that different parts of the face have different frequency compositions. In that case, some face parts' frequency domain synthesis is insufficient, resulting in the facial texture appearance with spots. Moreover, the reconstructed face is not very fine for some details like the eyes.

***Patch size for DFT***. We also explored the effect of different patch sizes on the reconstruction results. We show this effect by rendering the reconstruction results on a 2D plane and comparing the similarity between the rendered and input images. Structural similarity (SSIM) [59] is an indicator proposed to measure images' similarity, which can be applied to luminance, contrast, and structure. Peak Signal-to-Noise Ratio (PSNR) is defined as: *PSNR* = 10*∗*log_10_(*MAX*_*I*_^2^/*MSE*_〈*I*_*i*_, *I*_*r*_〉_), where MAX_*I*_^2^ is the maximum pixel value of the picture and MSE_〈*I*_*i*_, *I*_*r*_〉_ is the mean square error of the input image *I*_*i*_ and the rendered image *I*_*r*_. Learned perceptual image patch similarity (LPIPS) metric [60] uses the deep feature to measure the similarity of images. We also report SSIM, PSNR, and LPIPS between re-rendered images and original images under four patch sizes on the AFLW2000 dataset [17] and Now dataset [58] in Table 2. According to the result, we can see that the patch size of 4 × 4 shows the best performance.

## 5. Conclusion

We propose a spatio-frequency decoupled weak-supervision for 3D face reconstruction and build the weakly supervision by applying both spatial domain loss and frequency domain loss to enhance the reality of re-rendered facial images based on the reconstructed shape and texture. The key contribution is the designed spectrum-wise weighted Wing loss based on frequency loss on image patches, which narrows the gap between input and output in the frequency domain and captures inconspicuous frequency affecting reality. Experiments show the effectiveness of our method and comparable results with several state-of-the-art methods.

## Figures and Tables

**Table 1:** Quantitative evaluation on FS-lab benchmark categorized by pose angle.

Pose angle	0°			30°			60°		
Method	CD	MNE	CR	CD	MNE	CR	CD	MNE	CR
Deep3DFaceRec [13]	2.30	0.080	91.8	3.95	0.092	87.3	4.80	0.122	79.5
MGCNet [48]	3.45	0.085	92.7	3.91	0.092	90.1	3.65	0.090	83.2
DECA [25]	3.30	0.093	99.8	4.14	0.100	97.4	4.20	0.106	97.1
3DDFA_V2 [56]	3.05	0.093	95.2	3.41	0.096	93.8	3.82	0.096	88.1
PRNet [18]	2.94	0.132	92.5	3.40	0.125	90.1	3.74	0.121	85.1
FaceScape_deep [16]	2.40	0.086	96.7	7.27	0.124	87.7	3.87	0.108	90.5
UDL [20]	2.21	0.092	79.5	5.33	0.122	71.3	5.63	0.167	62.0
Ours	2.12	0.077	92.1	2.30	0.079	89.8	3.28	0.109	85.2

**Table 2:** Ablation studies of different patch sizes are important for the frequency loss.

Patch size	Indicator					
	AFLW2000			Now dataset		
	SSIM↑	PSNR↑	LPIPS↓	SSIM↑	PSNR↑	LPIPS↓
1 × 1	0.744	12.008	0.232	0.869	23.245	0.126
2 × 2	0.743	11.977	0.226	0.870	23.241	0.128
4 × 4	0.762	12.558	0.221	0.871	23.088	0.122
8 × 8	0.753	12.161	0.226	0.868	23.392	0.126

## Data Availability

Any data used to support the findings of this study are from previously reported studies and datasets, which have been cited.
